# Data on proteomic profiling of extracellular vesicles of Acholeplasma laidlawii strains with increased resistance to antibiotics of different classes - ciprofloxacin and tetracycline

**DOI:** 10.1016/j.dib.2020.106049

**Published:** 2020-07-19

**Authors:** Alexey Mouzykantov, Elena Medvedeva, Natalia Baranova, Olga Chernova, Vladislav Chernov

**Affiliations:** Kazan Institute of Biochemistry and Biophysics, FRC Kazan Scientific Center of RAS

**Keywords:** *Acholeplasma laidlawii*, Ciprofloxacin, Tetracycline, Resistance, Extracellular vesicles, Proteome, *1D SDS-PAGE,* LC-MS/MS

## Abstract

To elucidate the regularities of adaptation of the representatives of class Mollicutes to antimicrobials and to identify the promising targets for eradication of mycoplasma infections and contaminations the comparative analysis of the molecular basis of bacterial resistance to antibiotics of different classes is needed. Previously, we presented the data on the whole-genome sequences of *Acholeplasma laidlawii* strains with different susceptibility to ciprofloxacin (GenBank: LXYB00000000.1), tetracycline (GenBank: NELO00000000.2) and melittin (GenBank: NELN00000000.2) as well as the data on cell and extracellular vesicle proteomes of melittin-resistant *A. laidlawii* strain [1]. The lists of extracellular vesicle proteins secreted by *A. laidlawii* strains with the increased resistance to ciprofloxacin (PG8R_10_) and tetracycline (PG8R_Tet_) are presented here. The vesicle proteome profiles were obtained by 1D SDS-PAGE and liquid chromatography-mass spectrometry.

Specifications Table

**Table:** 

Subject	Microbiology
Specific subject area	Mollicute proteomics; vesiculome
Type of data	Table
How data were acquired	Instruments: 1D SDS-PAGE, mass spectrometer Maxis Impact (Bruker, Germany) equipped with a HPLC system Dionex Ultimate 3000 Series
Software: MASCOT program (Matrix Science)	
Data format	Raw
Parameters for data collection	*A. laidlawii* strains with differential susceptibility to antibiotics of different classes – tetracycline and ciprofloxacin
Description of data collection	The vesicles were obtained by ultracentrifugation; their purity was validated by PCR and TEM. The proteome profiling of vesicular proteins was performed using 1D SDS-PAGE and LC-MS/MS.
Data source location	Institution: KIBB FRC Kazan Scientific Center of RAS,
City/Town/Region: Kazan	
Country: Russia	
Data accessibility	The data are available with this article
Related research article	V.M. Chernov, A.A. Mouzykantov, N.B. Baranova, E.S. Medvedeva, T.Y. Grygorieva, M.V. Trushin, I.E. Vishnyakov, A.V. Sabantsev, S.N. Borchsenius, O.A. Chernova, Extracellular membrane vesicles secreted by mycoplasma *Acholeplasma laidlawii* PG8 are enriched in virulence proteins, J. Proteomics 110 (2014) 117–128. doi: 10.1016/j.jprot.2014.07.020.

**Value of the Data**

• These data show common and specific proteins transferred by extracellular vesicles of *A. laidlawii* strains with different susceptibility to tetracycline.
• These data show common and specific proteins transferred by extracellular vesicles of *A. laidlawii* strains with different susceptibility to ciprofloxacin.
• The presented data can be useful to the researchers studying molecular mechanisms of mycoplasma resistance to antimicrobials of different classes.
• The presented data can be useful to identify the promising targets for eradication of mycoplasma infections and contaminations.

## Data Description

**Fig. 1:**
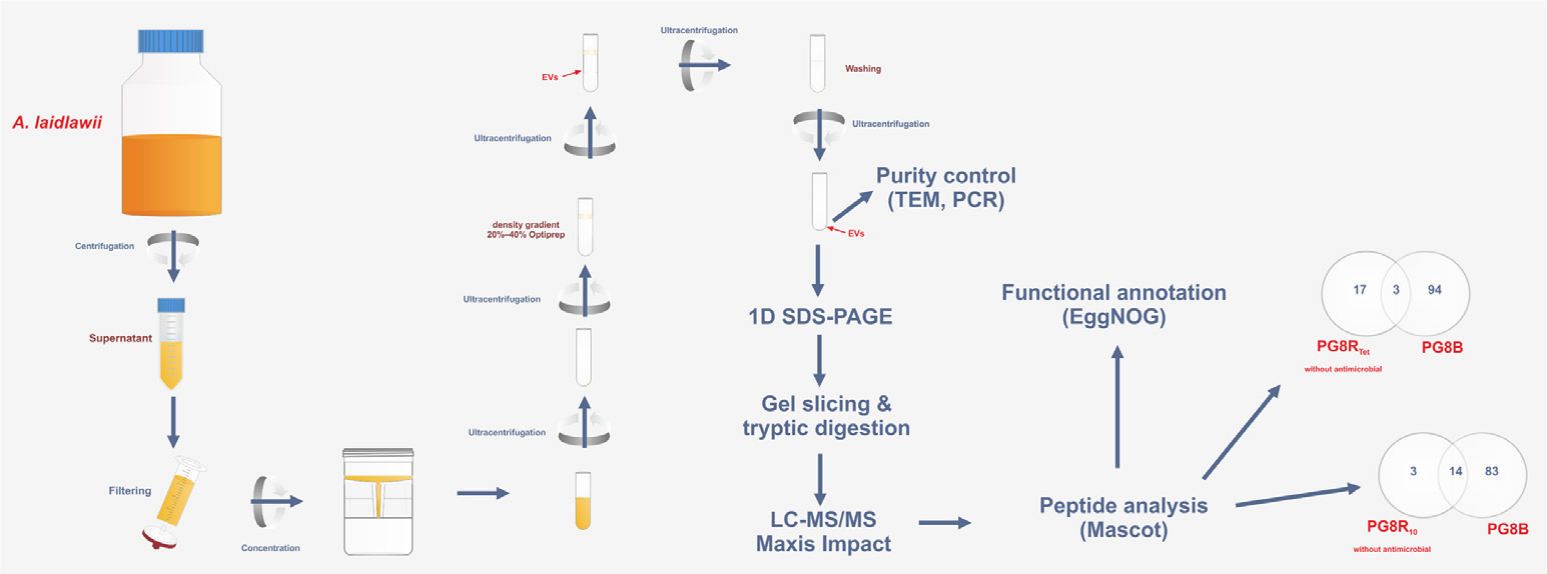
Study design.

**Fig. 2:**
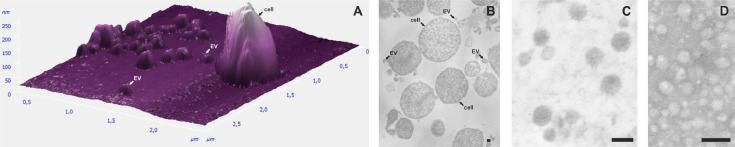
Micrographs of cells and EVs from *A. laidlawii* PG8 – the parent strain of PG8R_10_ and PG8R_Tet_, showing increased resistance to ciprofloxacin and tetracycline, respectively. Bar = 100 nm. A – AFM of *A.laidlawii* PG8 cells and EVs B – TEM of *A.laidlawii* PG8 cells and EVs С – TEM of EVs isolated from *A.laidlawii* PG8 D – negative-staining of EVs isolated from *A.laidlawii* PG8

**Fig. 3:**
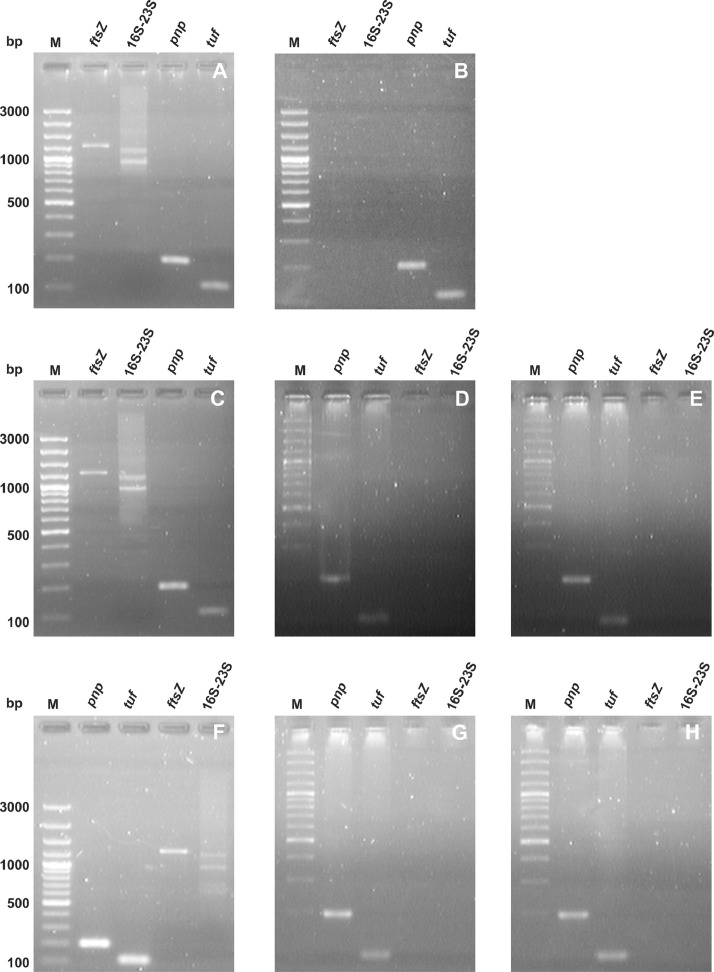
Electrophoregrams of the amplification products of the nucleotide sequences of *pnp, tufB, ftsZ* and 16S–23S rRNA gene intergenic spacer region of *Acholeplasma laidlawii*, which were obtained by PCR using the total DNA (as a template) isolated from the cells (A, C, F) and EVs (B, D, E, G, H) of *A.laidlawii* strains with increased resistance to ciprofloxacin and tetracycline - PG8R_10_ and PG8R_Tet,_ respectively. M - DNA Ladder Marker. A – DNA isolated from cells of *A. laidlawii* PG8 B – DNA isolated from EVs of *A. laidlawii* PG8 C – DNA isolated from cells of *A. laidlawii* PG8R_10_ (cultivation with ciprofloxacin) D – DNA isolated from EVs of *A. laidlawii* PG8R_10_ (cultivation with ciprofloxacin) E – DNA isolated from EVs of *A. laidlawii* PG8R_10_ (cultivation without ciprofloxacin) F – DNA isolated from cells of *A. laidlawii* PG8R_Tet_ (cultivation with tetracycline) G – DNA isolated from EVs of *A. laidlawii* PG8R_Tet_ (cultivation with tetracycline) H – DNA isolated from EVs of *A. laidlawii* PG8R_Tet_ (cultivation without tetracycline)

Extracellular vesicles were isolated from *A. laidlawii* strains with different sucseptibility to ciprofloxacin and tetracycline (PG8R_10,_ PG8R_Tet,_ PG8)(Fig. 1). The absence of bacterial cells in the preparations of vesicles of the strains was confirmed by TEM and/or PCR analysis with primers for marker nucleotide sequences of *A. laidlawii* extracellular vesicles (Fig. 2, Fig. 3). To characterize the vesicular proteome of the *A. laidlawii* strains with the increased resistance to ciprofloxacin (PG8R_10_) and tetracycline (PG8R_Tet_) 1D SDS-PAGE and LC-MS/MS were performed. As a result of the proteome analysis of extracellular vesicles secreted by tetracycline- and ciprofloxacin-resistant *A. laidlawii* strains (cultivation without/with antimicrobials), 20/21 and 17/19 proteins were identified, respectively. The proteins were isolated from pure vesicles and separated by one-dimensional electrophoresis. The protein bands were cleaved by trypsin and analyzed by mass-spectrometry. The lists of identified proteins of PG8R_Tet_ and PG8R_10_, their annotation information and functional classification are presented in Supplementary Table 1 and 2, respectively. The lists of the specific vesicular proteins derived from the strains with the increased resistance to ciprofloxacin - PG8R_10_ (MIC 20 mcg/ml) and tetracycline - PG8R_Tet_ (MIC 1 mcg/ml) compared to those from their parent strain - PG8B (MIC 0.5 mcg/ml and 0.08 mcg/ml respectively)[2] are summarized in Supplementary Table 3. The common vesicular protein derived from the resistant strains to antimicrobials of different classes (ciprofloxacin, tetracycline, melittin) was indicated in the Supplementary Table 3.

## Experimental Design, Materials and Methods

### Bacterial strains and culture conditions

The *A. laidlawii* cells were grown in a modified liquid Edward's medium (EM) at 37°C [2]. The ciprofloxacin- (PG8R_10_) and tetracycline-resistant (PG8R_Tet_) strains of *A. laidlawii* were grown in EM with antimicrobials (10 μg ml^−1^ ciprofloxacin and 0.64 μg ml^−1^ tetracycline, respectively) and without (one passage in EM) the antimicrobials.

### Isolation of extracellular vesicles

The isolation of the *A. laidlawii* extracellular vesicles was performed as described previously [1,3]. The cell-free vesicles were collected by ultracentrifugation (Beckman Coulter Optima™ MAX-E) and were purified by stepwise density gradient 20%–40% Optiprep ultracentrifugation. The resulting visual banding fraction was removed from the top, diluted and ultracentrifuged. The pellet obtained was dissolved in buffer supplemented with 1 mM PMSF. The absence of bacterial cells in the vesicle preparation was confirmed by transmission electron microscopy, plating on EM and PCR analysis with primers for marker nucleotide sequences of vesicle – 16S-23S rRNA gene intergenic spacer region ^−^, *ftsZ* ***^−^***, *pnp* ***^+^***, *tufB ^+^* [2].

### Transmission electron microscopy

TEM was performed as described previously [1]. Mycoplasma cells were collected by centrifugation 7,000 × g for 15 min. The cell and EV pellets were fixed with 2.5% glutaraldehyde solution prepared on phosphate buffered saline (0.1 M, pH 7.2) for 1.5 h at room temperature. Then the sample was kept in 1% OsO_4_ solution prepared in the same buffer supplemented with 25 mg / ml sucrose for 1.5 h. The pellets were washed with phosphate buffered saline (0.1 M, pH 7.2). Dehydration of the samples was carried out in increasing concentrations of alcohols as follows: rinsed in a 30% aqueous solution, then kept for 10 min 3 times at each stage in a 50%, 60%, 70%, 80% solution, in 90% - 4 times for 15 min, in pure acetone - 4 times for 15 min. After dehydration, the samples were kept in propylene oxide for 45 min after which epoxy resins (Epon 812, DDSA, MNA, DMP-30) and propylene oxide for 1 h at 37°C was performed in the following ratios: 1:2; 1:1; 2:1; then the sample was placed in a clean resin for 1–1.5 h at 45°C. Polymerization was carried out at temperatures of 37°C, 45°C and 60°C for 12 h. Ultrathin sections were prepared on a microtome LKB-III (Sweden), mounted on nickel grids, stained with an aqueous solution of uranyl acetate and lead citrate. Samples were analyzed on a JEM-1200EX electron microscope (Japan).

The negative-staining TEM was performed as described in [4]. The extracellular vesicles were placed on the carbon coated grid and incubated at 25°C for 10 s for adsorption of the proper amount of particles. Then excess solution was removed from the grid by blotting with a filter paper. Then to stain the particles a 2.5 µL of uranyl acetate solution was applied on the grid for 1 min. Excess of uranyl acetate solution was removed by blotting, and the grid was dried in air before imaging. A JEM-1200EX electron microscope was used to acquire a negative stain image.

### Atomic force microscopy

Atomic force microscopy (AFM) was performed according to [5]. To prepare samples for AFM *A. laidlawii* cell cultures were pelleted by centrifugation (10,000 g for 20 min) at room temperature. The pellets were then resuspended in phosphate buffered saline (0.1 mM, pH 7.2) and centrifuged again (10,000 g for 15 min). The resulting pellets were resuspended in phosphate-buffered saline (0.1 mM, pH 7.2). Top layer of mica (“Center for Advanced Technologies”, Russia) was removed by stationery tape. A cell suspension was applied to the surface of the mica in an amount of 5 μl; 5 min after application, the sample was washed three times with ddiH_2_O and dried. Examination of the samples was carried out at an atomic force microscope Solver P47H (NT-MDT, Russia). Cantilevers (fpN11S) with a needle radius of 10 nm (“Center for Advanced Technologies”, Russia) were used. The measurements were carried out in a semi-contact manner. For data processing, the Nova 1.0.26 RC1 software (NT-MDT, Russia) was used.

### Polymerase chain reaction

PCR primers were constructed in Litekh Research and Production Company (Moscow, Russia) using the nucleotide sequences of *A. laidlawii* genes (GenBank accession number NC_010163): *ftsZ* (5′-ggtttttggatttaacgatg-3′ and 5′-gcttccgcctcttttattt-3′), 16S–23S rRNA gene intergenic spacer region (5′-ggaggaaggtggggatgacgtcaa-3′ and 5′-ccttaggagatggtcctcctatcttcaaac-3′), *pnp* (5′-aagcccattgcgatacctgc-3′ and 5′-ggtgctttaggagaacgtgct-3′), *tufB* (5′-ccaggtcacgctgactatgtt-3′ and 5′-acgagtttgtggcattggac-3′). PCR was performed in the following regime: for *ftsZ* 95°С, 3 min; (95°С, 30 s; 52°С, 90 s; 72°С, 60 s) (30 cycles); 72°С, 10 min; for 16S–23S rRNA gene intergenic spacer region, 95°С, 3 min (95°С, 5 s; 63°С, 5 s; 72°С, 20 s) (30 cycles); 72°С, 5 min; for *pnp* and *tufB* 95°С, 3 min (95°С, 5 s; 52°С 5 s; 72°С 5 s) (35 cycles); 72°С 5 min.

### SDS-PAGE and in-gel digestion

Preparation of vesicular proteins, their separation using 1D SDS-PAGE and tryptic digestion were performed according to [3,6]. The vesicles were mixed with 6x sample buffer (375 mM Tris-HCl pH 6.8, 60% glycerol, 10% SDS, 0.6 M DTT, and 0.01% bromophenol blue) and incubated at 95°C for 5 min. Samples were run in 12% SDS-PAGE mini-gel until bromophenol blue front was 1 cm from the bottom. The gel was subsequently stained with Coomassie G-250. The protein fractions were cut out from the gel and washed for 15 min in ddiH_2_O and then in a solution containing a 1:1 mixture of acetonitrile and 200 mM NH_4_HCO_3_ at 50°С for 30 min. Protein reduction and alkylation was performed using a 10 mM DTT and 50 mM iodoacetamide respectively [6]. The gels were incubated in acetonitrile, dried and incubated in trypsin Gold (Promega, USA) solution for 60 min at 4°C. The tryptic digestion was performed overnight at 37°C. To extract the peptides from the gel, a 0.5% TFA solution was added to each tube and the tubes were incubated in an ultrasonic bath for 10 min. The extract was transferred to a clean tube and dried in a centrifugal evaporator. Samples were analyzed with mass-spectrometry.

### LC-MS/MS analysis

LC-MS/MS analysis and identification of proteins were as described previously [2]. The tryptic digested samples were dissolved in a mixture of 98.9% water, 1% methanol, 0.1% formic acid (v/v), loaded to an Acclaim PepMap RSLC column (Thermo Fisher Scientific, USA) and eluted for 5 h, increasing the concentration of a mixture of 99.9% acetonitrile and 0.1% formic acid (v/v) from 2 to 60%. Mass spectra were obtained on a mass spectrometer Maxis Impact (Bruker, Germany) equipped with a HPLC system Dionex Ultimate 3000 Series (Thermo Fisher Scientific, USA). The MS1 mass spectra were obtained as follows: detection of molecular ions was performed in the range was 300–2000 m/z, with a signal accumulation time of 250 ms. To obtain MS2 spectra, ions with a signal-to-noise ratio of at least 400 and a charge from 2 to 5 were selected. Ion detection was performed in the range of 200-2000 m/z, with a signal accumulation time of 50 ms for each parent ion. The measurement accuracy was 0.6 Da. The resulting MS/MS spectra were analyzed using the MASCOT program [7]. Protein identification was considered reliable when at least two peptides with different amino acid sequences with a pepscore value ≥ 15 were detected.

### Analysis of amino acid sequences *in silico*

The functional annotation of each protein was performed according to EggNOG (http://eggnog5.embl.de/)[8].

## Ethics Statemen

The work involved bacteria, but did not involve the use of human subjects or animals.

## Declaration of Competing Interest

The authors declare that they have no known competing financial interests or personal relationships which have, or could be perceived to have, influenced the work reported in this article.

## Appendix Supplementary materials

Image, application 1

## References

[1] Medvedeva E.S., Mouzykantov A.A., Baranova N.B., Dramchini M.A., Chernova O.A., Chernov V.M. (2019). Data on proteomic profiling of cells and extracellular vesicles of the melittin-resistant *Acholeplasma laidlawii* strain. Data Brief..

[2] Chernov V.M., Mouzykantov A.A., Baranova N.B., Medvedeva E.S., Grygorieva T.Y., Trushin M.V., Vishnyakov I.E., Sabantsev A.V., Borchsenius S.N., Chernova O.A. (2014). Extracellular membrane vesicles secreted by mycoplasma *Acholeplasma laidlawii* PG8 are enriched in virulence proteins. J. Proteomics.

[3] Lee E.-Y., Choi D.-Y., Kim D.-K., Kim J-W, Park J.O., Kim S., Kim S.-H., Desiderio D.M., Kim Y.-K., Kim K.-P., Gho Y.S. (2009). Gram-positive bacteria produce membrane vesicles: proteomics-based characterization of *Staphylococcus aureus*-derived membrane vesicles. Proteomics.

[4] Sharpe S.W., Kuehn M.J., Mason K.M. (2011). Elicitation of epithelial cell-derived immune effectors by outer membrane vesicles of nontypeable *Haemophilus influenzae*. Infect. Immun..

[5] Braga P.C., Ricci D. (2004). Imaging bacterial shape, surface, and appendages before and after treatments with antibiotics. Methods Mol. Biol..

[6] Gundry R.L., White M.Y., Murray C.I., Kane L.A., Fu Q., Stanley B.A., Van Eyk J.E. (2009). Preparation of proteins and peptides for mass spectrometry analysis in a bottom-up proteomics workflow. Curr. Protoc. Mol. Biol..

[7] Brosch M., Yu L., Hubbard T., Choudhary J. (2009). Accurate and sensitive peptide identification with Mascot percolator. J. Proteome. Res..

[8] Huerta-Cepas J., Szklarczyk D., Forslund K., Cook H., Heller D., Walter M.C., Rattei T., Mende D.R., Sunagawa S., Kuhn M., Jensen L.J., von Mering C., Bork P. (2016). eggNOG 4.5: a hierarchical orthology framework with improved functional annotations for eukaryotic, prokaryotic and viral sequences. Nucl. Acids. Res..

